# Comparative analysis of the quality of life regarding patients who underwent hip replacement in public versus private hospitals in Hungary

**DOI:** 10.1038/s41598-024-60720-4

**Published:** 2024-05-01

**Authors:** Luca Fanni Kajos, Bálint Molics, Péter Than, Gyula Gőbel, Diána Elmer, Dalma Pónusz-Kovács, Tímea Csákvári, Bettina Kovács, Lilla Horváth, József Bódis, Imre Boncz

**Affiliations:** 1 Institute for Health Insurance, Faculty of Health Sciences, University of Pécs, Pécs, 7621 Hungary; 2 Doctoral School of Health Sciences, Faculty of Health Sciences, University of Pécs, Pécs, 7621 Hungary; 3 National Laboratory on Human Reproduction, Pécs, 7624 Hungary; 4 Institute of Physiotherapy and Sport Science, Faculty of Health Sciences, University of Pécs, Pécs, 7621 Hungary; 5 Department of Orthopaedics, Clinical Centre, Medical School, University of Pécs, Pécs, 7632 Hungary; 6 Da Vinci Private Clinic, Pécs, 7635 Hungary; 7 Department of Obstetrics and Gynaecology, Clinical Centre, Medical School, University of Pécs, Pécs, 7624 Hungary

**Keywords:** Quality of life, Hip replacement, Public and private hospital, OHS, SF-36, Hungary, Quality of life, Osteoarthritis, Orthopaedics, Cartilage, Osteoarthritis, Public health

## Abstract

The study aimed to investigate the impact of hip replacement surgery on the quality of life and to compare the outcomes by sociodemographic and surgical data in Hungarian public and private hospitals. Patients were selected at the Department of Orthopaedics (Clinical Centre, University of Pécs) and at the Da Vinci Private Clinic in Pécs. Patients completed the SF-36 and Oxford Hip Score (OHS) questionnaires before the surgery, 6 weeks and 3 months later. We also evaluated socio-demographic data, disease and surgical conditions. The research involved 128 patients, 60 patients in public, 68 patients in private hospital. Despite the different sociodemographic characteristics and surgical outcomes of public and private healthcare patients, both groups had significantly improved the quality of life 3 months after hip replacement surgery measured by OHS and SF-36 physical health scores (*p* < 0.001). In the mental health score, only the patients of the private health sector showed a significant improvement (*p* < 0.001). The extent of improvement did not differ between the two healthcare sectors according to the OHS questionnaire (*p* = 0.985). While the SF-36 physical health score showed a higher improvement for public patients (*p* = 0.027), the mental health score showed a higher improvement for private patients (*p* = 0.015).

## Introduction

The hip joint is associated with several joint pathologies, which are related to pain in the affected area, limited movement, and the deterioration regarding the quality of life. For the surgical treatment of these pathologies (for example osteoarthritis, avascular necrosis of the femoral head, dysplastic hip, fracture), if the patient’s physical condition allows, endoprosthesis implantation is used, which has been proven to reduce joint pain, improve the range of motions, and also has a positive effect on the individual’s quality of life and leads to patient satisfaction^1,2^.

Hip replacement surgery is one of the most popular, cost-effective, and successful surgical procedures^3,4^. The number of surgeries performed is gradually increasing with the aging of the population, according to current estimates, it is more than 1,000,000 surgeries worldwide annually^5–7^. The key to the surgery’s popularity lies in the significant improvement in quality-of-life indicators, in addition to the positive change in functional status. There is a large amount of literature that proves the impact of hip replacement surgery on health-related quality of life (HRQoL) in the short and long term. Studies are increasingly focusing on measuring patient-reported outcomes (PROs), especially for patients undergoing joint replacement surgery, where the primary outcome is pain reduction and improved quality of life^8^. The results obtained during quality-of-life measurement contribute to the improvement considering the quality of care and the identification of the strengths and weaknesses of the care. They play an increasingly important role in evaluating the efficiency of healthcare and individual health interventions, in performance assessment, for both clinical decision-makers and healthcare providers^1,2,9^.

The role of the minimally invasive technique is still controversial, and the effect on surgical results is a matter of debate. Based on international literature, this procedure is associated with less pain, faster postoperative rehabilitation, less muscle and soft tissue damage, reduced blood loss, a more aesthetic appearance and a shorter hospital stay, but according to recently published studies, it had no relevant advantages compared to the traditional procedure^6,10–13^. Also, the examination between explorations did not show significant differences in functional results and quality of life^14^. Although many international research studies the effects of hip replacement surgery on the quality of life, little information is available the differences between public and private hospitals.

The purpose of our present research is to examine the effects of hip replacement surgery on the quality of life with a 3-month follow-up and to compare quality-of-life indicators depending on sociodemographic and surgical data in the public and private healthcare systems in Hungary. The main novelty of our research is that the results are also examined from the side of the Hungarian public and private hospitals, where the selected institutions operate with different surgical procedures and techniques.

## Methods

### Study design

The type of research is a quantitative, prospective, longitudinal follow-up study. The investigation took place between April 2019 and March 2020. The examination was carried out at the Department of Orthopaedics of the Clinical Centre of the University of Pécs in the case of public healthcare, and at the Da Vinci Private Clinic in Pécs in the case of private healthcare. While the tertiary level university public hospital is exclusively publicly funded, the private institution performs only surgeries financed by the patient and do not have public financing. Detailed description of the Hungarian healthcare system can be found elsewhere^15–17^.

### Patients

The examined patients were patients who underwent hip replacement surgery at the Department of Orthopaedics of the Clinical Centre of the University of Pécs and at the Da Vinci Private Clinic in Pécs, who were selected using a simple convenience sampling technique. The heads of the institutes gave previously written permission. The subjects met the conditions of the study according to the inclusion and exclusion criteria and received verbal and written information at the beginning of the survey. By signing the information statement and the consent statement, they agreed to participate in the study, consented to the anonymous publication of their data, and full participation in the surveys.

According to the inclusion criteria, the participants reached the age of 18, they agreed to participate in the research, underwent hip replacement surgery and were able to fill in the questionnaires independently. An exclusionary reason was if the indication for surgery was a malignant tumour or an acute fracture, as well as neurological, cognitive and/or psychological disorders that hindered the understanding of the questions in the questionnaire and filling in the questionnaire. During the evaluation, we did not consider patients who did not complete a follow-up questionnaire, as well as those who died during the study period.

### Survey

During the survey patients filled in one self-edited and two internationally validated questionnaires, the first time (before surgery) in person, and then during the follow-up period by telephone, email or by post (in pre-stamped and addressed envelopes). Our questionnaire survey on the quality of life took place at the following times: before surgery, 6 weeks and 3 months after surgery. The first quality-of-life questionnaire survey was about the preoperative health status (the health status of the 4 weeks before the operation). At the same time, we carried out the survey of socioeconomic factors and disease-related data with the self-made questionnaire. At the time of discharge from the hospital, we collected information related to the surgery by questioning the patients and checking the data from the patient documentation under medical supervision.

### Measurement tools

The Short-form 36 Health Status Questionnaire (SF-36) and the Oxford Hip Score (OHS) were used to examine the quality of life. The SF-36 questionnaire is a scale for examining the general quality of life, which can be used from the age of 14. As the survey’s name suggests, it examines patients’ perceptions of their own health in 36 simple multiple-choice questions. Within this, 8 different groups of questions can be distinguished: physical activity (PF), role limitation due to physical problems (RP), physical pain (BP), general health perception (GH), vitality (VT), social activity (SF), role limitation due to emotional problems (RE) and general mental health (MH). The evaluation can be scored from 0 to 100 for these 8 dimensions based on the patient’s answers. A score of 0 represents the worst quality of life score and a score of 100 represents the best quality of life score available. The scoring can be further divided into two groups to determine the physical health (PCS) and mental health (MCS) status of the respondent^18,19^. The test is also validated in the Hungarian version, and Hungarian normal values for the healthy population are also available^20^. One of the most commonly used disease-specific questionnaires for hip and knee joints is the Oxford Hip Score, which uses 12 simple multiple-choice questions to assess the severity, nature, duration of pain, lameness, and limitation of daily activities. Each question is scored from 0 to 4 points per answer, with a maximum score of 48 points. The highest score means that the patient has a better joint condition^21^. The Hungarian-language questionnaire was used in several earlier studies in Hungary^22–26^. We also performed a previous study with a translated questionnaire to assess the applicability and validity of OHS questionnaire. A Hungarian translation of the questionnaire was used during the study (ID: 7839- PTE 2019, 07/06/2019)^22^.

### Statistical analysis

Descriptive statistical analysis, paired T-test, independent sample T-test, Pearson’s chi-squared test, Mann–Whitney U test, Wilcoxon test, one-way and mixed ANOVA, and Kruskal–Wallis test were performed to determine the results of the study at the 95% significance level (*p* < 0.05). Pearson’s chi-squared test was used to compare sociodemographic and surgical data between healthcare sectors (Tables 1 and 2). Comparisons of quality-of-life scores between healthcare sectors over the study period were made using paired T-test and independent sample T-test, where a normal distribution was observed. Where normality test (Shapiro–Wilk test) indicated that a normal distribution was not fulfilled, Mann–Whitney U test and Wilcoxon test were used. One-way ANOVA and Kruskal–Wallis test were also applied for this analysis (Table 3). To compare sociodemographic data and surgical data with the quality-of-life scores (OHS) of patients in each healthcare sector over the study period, we also conducted one-way ANOVA, Kruskal–Wallis test and mixed ANOVA to complement our statistical analysis (Tables 4 and 5). The association between quality-of-life outcomes, socioeconomic data and type of hospital was analysed using multivariate linear regression (Table 6). The data was analysed using Microsoft Excel 2016 and the IBM SPSS 24 statistical program.

**Table 1:** Comparison of sociodemographic characteristics.

		Public hospital		Private hospital		*p* value
		N	%	N	%	
Participants	Number of persons	60	100%	68	100%	
	Number of persons (male)	22	36.67%	30	44.12%	
	Number of persons (female)	38	63.33%	38	55.88%	
Age	Mean age (SD)	66.05 (7.43)		63.06 (10.58)		
	< 45 years	0	0%	5	7.35%	
	46–64 years	19	31.67%	29	42.65%	
	> 65 years	41	68.33%	34	50.0%	0.036*
BMI	Underweight	0	0%	0	0%	
	Normal	14	23.33%	16	23.53%	
	Overweight	24	40.0%	24	35.29%	
	Obesity	22	36.67%	28	41.18%	
Residence	Village	20	33.3%	12	17.65%	0.041*
	City	25	41.67%	23	33.82%	
	County seat	15	25.0%	26	38.24%	
	Capital	0	0%	5	7.35%	
	Other (municipality)	0	0%	2	2.94%	
Education	Primary	13	21.67%	2	2.94%	0.001*
	Secondary	34	56.67%	28	41.18%	
	Higher	13	21.67%	38	55.88%	< 0.001*
Marital status	Married	35	58.33%	42	61.76%	
	In a relationship	3	5.0%	7	10.29%	
	Single	2	3.33%	5	7.35%	
	Divorced	9	15.0%	6	8.82%	
	Widow	11	18.33%	8	11.76%	
Occupation	Intellectual	5	8.33%	27	39.71%	< 0.001*
	Light physical	4	6.67%	6	8.82%	
	Hard physical	3	5.0%	3	4.41%	
	Pensioner	45	75.0%	32	47.06%	0.001*
	Other (unemployed, disabled)	3	5.0%	0	0%	
Employee status	Government employee	6	10.0%	5	7.35%	
	Privately employed	6	10.0%	12	17.65%	
	Entrepreneur	0	0%	23	33.82%	< 0.001*
	Other (retired, unemployed, disabled)	48	80.0%	28	41.18%	< 0.001*

*Asterisked data indicate statistically significant results. *SD* standard deviation.

**Table 2:** Comparison of surgical characteristics.

		Public hospital		Private hospital		*p* value
		N	%	N	%	
Indication for surgery	Primary coxarthrosis	49	81.67%	58	85.29%	
	Avascular necrosis of the femoral head	5	8.33%	4	5.88%	
	Dysplastic hip (secondary coxarthrosis)	3	5.0%	6	8.82%	
	Revision	3	5.0%	0	0%	
Surgical procedure	Traditional	60	100%	0	0%	< 0.001*
	Minimally invasive	0	0%	68	100%	< 0.001*
Type of exploration	Anterior	0	0%	68	100%	< 0.001*
	Posterior	1	1.67%	0	0%	
	Anterolateral	59	98.33%	0	0%	< 0.001*
Type of fixation	Cemented	22	36.67%	5	7.35%	< 0.001*
	Non-cemented	36	60.0%	63	92.65%	< 0.001*
	Hybrid	2	3.33%	0	0%	
Type of anaesthesia	Anaesthesia	28	46.67%	11	16.18%	< 0.001*
	Narcotization	26	43.33%	24	35.29%	
	Anaesthesia + Narcotization	1	1.67%	20	29.41%	< 0.001*
	Narcotization + stupor	5	8.33%	13	19.12%	
Drain	Yes	60	100%	17	25.0%	< 0.001*
	No	0	0%	51	75.0%	< 0.001*
Blood transfusion	Yes	18	30.0%	0	0%	< 0.001*
	No	41	68.33%	68	100%	< 0.001*
	No information	1	1.67%	0	0%	

*Asterisked data indicate statistically significant results.

**Table 3:** Change in quality-of-life score during the study period.

		Public hospital	Private hospital	*p* value
		Score (n = 60)	Score (n = 68)	
Oxford Hip Score	Before surgery (SD)	16.60 (8.47)	22.78 (10.37)	< 0.001*
	6th week (SD)	28.67 (8.55)	35.01 (7.98)	< 0.001*
	3rd month (SD)	34.68 (8.89)	40.85 (7.22)	< 0.001*
	Improvement within the group	< 0.001*	< 0.001*	
	Improvement between groups	*p* = 0.985		
SF-36 physical health	Before surgery (SD)	26.09 (16.76)	37.32 (20.84)	< 0.001*
	6th week (SD)	44.31 (17.37)	64.89 (20.17)	< 0.001*
	3rd month (SD)	56.68 (21.51)	77.35 (19.75)	< 0.001*
	Improvement within the group	< 0.001*	< 0.001*	
	Improvement between groups	*p* = 0.027*		
SF-36 mental health	Before surgery (SD)	66.85 (23.32)	68.63 (20.43)	
	6th week (SD)	67.25 (23.98)	81.09 (19.05)	< 0.001*
	3rd month (SD)	73.86 (22.38)	86.18 (16.31)	< 0.001*
	Improvement within the group	*p* = 0.075	< 0.001*	
	Improvement between groups	*p* = 0.015*		

*Asterisked data indicate statistically significant results. *SD* standard deviation.

**Table 4:** Comparison of sociodemographic characteristics and the Oxford Hip Score.

		Public hospital			Private hospital				
		OHS score (before surgery)	OHS score (3 months)	p value within group	OHS score (before surgery)	OHS score (3 months)	*p* value within group		*p* value between groups
Sex	Male (SD)	19.55 (9.81)	36.41 (7.59)	< 0.001*	28.6 (9.48)	41.93 (7.53)	< 0.001*		0.234
	Female (SD)	14.89 (7.19)	33.68 (9.52)	< 0.001*	18.18 (8.66)	40 (6.95)	< 0.001*		0.234
	*p* value	0.039*	0.256	0.548	< 0.001*	0.276	0.001*		0.096
Age	< 45 years (SD)	–	–	–	22 (7.11)	40.8 (5.76)	0.001*		–
	46–64 years (SD)	16.32 (8.49)	34.63 (7.37)	< 0.001*	24.97 (9.06)	42.93 (5.39)	< 0.001*		0.773
	> 65 years (SD)	16.73 (8.57)	34.71 (9.6)	< 0.001*	21.03 (11.61)	39.09 (8.38)	< 0.001*		0.853
	*p* value	0.861	0.976	0.919	0.323	0.108	0.987		0.919
BMI	Underweight	–	–	–	–	–	–		–
	Normal (SD)	17.57 (7.17)	34.64 (7.40)	< 0.001*	18.81 (10.17)	44.00 (4.46)	< 0.001*		0.035*
	Overweight (SD)	16.17 (10.72)	34.50 (9.11)	< 0.001*	26.21 (10.19)	41.67 (7.86)	< 0.001*		0.408
	Obesity (SD)	16.45 (6.58)	34.91 (9.86)	< 0.001*	22.11 (10.03)	38.36 (7.26)	< 0.001*		0.463
	*p* value	0.885	0.988	0.937	0.077	0.033*	0.006*		0.070
Residence	Village (SD)	15.15 (6.44)	33.2 (8.45)	< 0.001*	21 (12.14)	38.33 (7.04)	< 0.001*		0.834
	City (SD)	18 (9.19)	35.08 (9.26)	< 0.001*	22.13 (10.93)	43.43 (4.59)	< 0.001*		0.189
	County seat (SD)	16.2 (9.74)	36 (9.16)	< 0.001*	23.58 (10.04)	40.73 (7.35)	< 0.001*		0.529
	Capital (SD)	–	–	–	24.8 (6.14)	40 (9.06)	0.021*		–
	Other (municipality) (SD)	–	–	–	25.5 (13.44)	30 (18.39)	0.421		–
	*p* value	0.530	0.634	0.786	0.926	0.056	0.189		0.340
Education	Primary (SD)	16.23 (6.23)	29.85 (11.48)	< 0.001*	8.5 (6.36)	46 (2.83)	0.042*		0.004*
	Secondary (SD)	16.56 (9.98)	35.06 (8.18)	< 0.001*	22.21 (11.68)	38.61 (8.62)	< 0.001*		0.499
	Higher (SD)	17.08 (6.30)	38.54 (5.58)	< 0.001*	23.95 (9.02)	42.24 (5.72)	< 0.001*		0.309
	*p* value	0.968	0.039*	0.231	0.112	0.075	0.021*		0.010*
Marital status	Married (SD)	17.97 (9.70)	36.6 (7.72)	< 0.001*	22.45 (9.81)	40.95 (7.02)	< 0.001*		0.961
	In a relationship (SD)	17.67 (3.22)	35.67 (9.45)	0.124	28.43 (12.87)	42.14 (9.08)	0.008*		0.556
	Single (SD)	11 (2.83)	41 (2.83)	No data^a^	18.4 (8.59)	45.2 (2.68)	0.002*		0.644
	Divorced (SD)	15.33 (6.36)	31 (7.30)	< 0.001*	24.67 (10.11)	38.17 (3.71)	0.017*		0.632
	Widow (SD)	14 (6.80)	30.18 (12.11)	0.002*	20.88 (12.43)	38.5 (9.90)	0.006*		0.815
	*p* value	0.558	0.130	0.612	0.491	0.454	0.210		0.966
Occupation	Intellectual (SD)	21.2 (8.82)	35.2 (7.82)	0.071	24.78 (9.31)	41.63 (6.32)	< 0.001*		0.593
	Light physical (SD)	10.25 (7.68)	42.75 (3.86)	0.009*	24.33 (4.93)	41.33 (7.50)	0.002*		0.026*
	Hard physical (SD)	13 (5.57)	34 (8.0)	0.098	28.33 (11.93)	45 (3.0)	0.097		0.657
	Pensioner (SD)	16.38 (8.40)	34.16 (9.31)	< 0.001*	20.28 (11.53)	39.72 (8.12)	< 0.001*		0.528
	Other (unemployed, disabled) (SD)	24.33 (6.81)	31.67 (8.08)	0.069	–	–	–		–
	*p* value	0.144	0.440	0.049*	0.281	0.558	0.804		0.130
Employee status	Government employee (SD)	20.33 (8.96)	33.33 (8.78)	0.086	19.6 (6.88)	40 (8.03)	0.026*		0.411
	Privately employed (SD)	11.17 (6.59)	38.67 (8.14)	0.003*	23.5 (10.27)	42.75 (4.56)	< 0.001*		0.130
	Entrepreneur (SD)	–	–	–	26.22 (9.64)	41.3 (7.91)	< 0.001*		–
	Other (retired, unemployed, disabled) (SD)	16.81 (8.45)	34.35 (9.03)	< 0.001*	20.21 (11.04)	39.82 (7.57)	< 0.001*		0.436
	*p* value	0.161	0.502	0.080	0.190	0.678	0.424		0.146

*Asterisked data indicate statistically significant results. ^a^The correlation and t cannot be computed because the standard error of the difference is 0. *SD* standard deviation.

**Table 5:** Comparison of surgical characteristics and the Oxford Hip Score.

		Public hospital			Private hospital			*p* value between groups
		OHS score (before surgery)	OHS score (3 months)	*p* value within group	OHS score (before surgery)	OHS score (3 months)	*p* value within group	
Indication for surgery	Primary coxarthrosis (SD)	16.39 (7.18)	34.12 (9.13)	< 0.001*	22.17 (10.32)	40.83 (7.29)	< 0.001*	0.661
	Avascular necrosis of the femoral head (SD)	11.4 (7.02)	34.6 (9.99)	0.012*	23.5 (10.50)	39 (8.98)	0.099	0.384
	Dysplastic hip (secondary coxarthrosis) (SD)	11.33 (6.81)	41.67 (4.16)	0.021*	28.17 (10.91)	42.33 (6.19)	0.002*	0.010*
	Revision (SD)	34 (13.12)	37 (5.0)	0.801	−	−	−	−
	*p* value	0.001	0.533	0.024*	0.405	0.778	0.544	0.066
Surgical procedure	Traditional (SD)	16.6 (8.47)	34.68 (8.89)	< 0.001*	−	−	−	−
	Minimally invasive (SD)	−	−	−	22.78 (10.37)	40.85 (7.22)	< 0.001*	−
	*p* value	^−^	−	−	−	−	−	−
Type of exploration	Anterior (SD)	−	−	−	22.78 (10.37)	40.85 (7.22)	< 0.001*	−
	Anterolateral (SD)	16.56 (8.54)	34.53 (8.88)	< 0.001*	−	−	−	−
	Posterior	19	44	No data.^a^	−	−	−	−
	*p* value	0.778	0.294	0.560	−	−	−	−
Type of fixation	Cemented (SD)	13.95 (5.40)	32 (10.43)	< 0.001*	19.4 (11.24)	40.2 (7.79)	0.001*	0.558
	Non-cemented (SD)	18.83 (9.29)	35.89 (7.53)	< 0.001*	23.05 (10.34)	40.9 (7.24)	< 0.001*	0.736
	Hybrid (SD)	5.5 (4.95)	42.5 (7.78)	0.152	−	−	−	−
	*p* value	0.015*	0.121	0.065	0.453	0.835	0.551	0.742
Type of anaesthesia	Anaesthesia (SD)	16.5 (9.09)	34.64 (10.05)	< 0.001*	21.82 (8.97)	41.64 (6.87)	< 0.001*	0.710
	Narcotization (SD)	15.62 (7.80)	33.42 (8.06)	< 0.001*	21.58 (10.14)	41.5 (7.26)	< 0.001*	0.508
	Anaesthesia + Narcotization (SD)	22	36	No data.^a^	23.85 (10.82)	40.4 (7.13)	< 0.001*	0.822
	Narcotization + stupor (SD)	21.2 (9.04)	41.2 (3.90)	0.009*	24.15 (11.95)	39.69 (8.18)	< 0.001*	0.354
	*p* value	0.536	0.364	0.968	0.842	0.870	0.538	0.800
Drain	Yes (SD)	16.6 (8.47)	34.68 (8.89)	< 0.001*	22.76 (11.31)	40.59 (6.91)	< 0.001*	0.938
	No (SD)	−	−	−	22.78 (10.15)	40.94 (7.39)	< 0.001*	−
	*p* value	−	−	−	0.995	0.863	0.911	−
Blood transfusion	Yes (SD)	15.72 (12.49)	32.22 (10.43)	0.001*	−	−	−	−
	No (SD)	16.83 (6.18)	35.68 (8.14)	< 0.001*	22.78 (10.37)	40.85 (7.22)	< 0.001*	0.692
	*p* value	0.649	0.174	0.490	−	−	−	−

*Asterisked data indicate statistically significant results. ^a^The correlation and t cannot be computed because the sum of case weights is less than or equal to 1.—t cannot be computed because at least one of the groups is empty. *SD* standard deviation.

**Table 6:** Evaluation of the association between quality-of-life scores (OHS), socioeconomic data and type of hospital (multivariate linear regression, n = 128).

Oxford Hip Score										
Predictors	6th week					3rd month				
	Unstandardized Coefficients		Standardized Coefficients	t	Sig	Unstandardized Coefficients		Standardized Coefficients	t	Sig
	B	Std. Error	Beta			B	Std. Error	Beta		
Constant (Intercept)	49.780	7.560		6.585	<0.001*	43.075	7.080		6.084	<0.001*
Sex (reference: male)	− 2.651	1.655	− 0.148	− 1.602	0.112	− 2.102	1.550	− 0.121	− 1.356	0.178
Age group (reference: > 65 years)										
< 45 years	− 3.081	4.660	− 0.068	− 0.661	0.510	− 3.765	4.364	− 0.085	− 0.863	0.390
46–64 years	− 0.075	2.490	− 0.004	− 0.030	0.976	0.174	2.332	0.010	0.075	0.941
BMI (reference: normal)										
Overweight	− 2.944	2.113	− 0.162	− 1.393	0.166	− 0.926	1.979	− 0.052	− 0.468	0.641
Obesity	− 2.742	2.072	− 0.152	− 1.324	0.189	− 2.667	1.940	− 0.152	− 1.375	0.172
Residence (reference: capital)										
Village	− 6.955	4.189	− 0.343	− 1.660	0.100	− 0.831	3.923	− 0.042	− 0.212	0.833
City	− 7.027	4.068	− 0.388	− 1.728	0.087	4.113	3.810	0.233	1.080	0.283
County seat	− 7.299	3.968	− 0.388	− 1.840	0.069	2.148	3.716	0.117	0.578	0.565
Other (municipality)	− 12.544	6.949	− 0.177	− 1.805	0.074	− 8.628	6.508	− 0.125	− 1.326	0.188
Education (reference: primary)										
Secondary	3.500	2.687	0.199	1.302	0.196	1.248	2.517	0.073	0.496	0.621
Higher	3.097	2.981	0.173	1.039	0.301	4.588	2.792	0.263	1.643	0.103
Marital status (reference: single)										
Married	− 7.443	3.456	− 0.415	− 2.153	0.034*	− 5.799	3.237	− 0.332	− 1.791	0.076
In a relationship	− 8.695	4.303	− 0.266	− 2.021	0.046*	− 6.262	4.030	− 0.196	− 1.554	0.123
Divorced	− 10.934	3.933	− 0.401	− 2.780	0.006*	− 10.305	3.684	− 0.387	− 2.797	0.006*
Widow	− 10.539	4.308	− 0.427	− 2.446	0.016*	− 10.039	4.035	− 0.417	− 2.488	0.014*
Occupation (reference: light physical)										
Intellectual	− 2.314	3.175	− 0.114	− 0.729	0.468	− 5.185	2.973	− 0.262	− 1.744	0.084
Hard physical	− 4.083	4.405	− 0.098	− 0.927	0.356	0.019	4.126	0.000	0.005	0.996
Pensioner	− 4.522	4.721	− 0.252	0.958	0.340	− 8.454	4.421	− 0.484	− 1.912	0.059
Other (unemployed, disabled)	− 8.698	6.393	− 0.150	− 1.361	0.177	− 12.817	5.987	− 0.227	− 2.141	0.035*
Employee status (reference: government employee)										
Privately employed	− 0.627	3.235	− 0.025	− 0.194	0.847	2.697	3.029	0.110	0.890	0.375
Entrepreneur	− 0.320	3.298	− 0.014	− 0.097	0.923	0.293	3.089	0.013	0.095	0.925
Other (retired, unemployed, disabled)	− 0.219	4.199	− 0.012	− 0.052	0.958	5.979	3.933	0.343	1.520	0.131
Type of hospital (reference: public)	3.968	1.864	0.226	2.129	0.036*	5.007	1.746	0.292	2.868	0.005*

*Asterisked data indicate statistically significant results. Predictors 6th week: R = 0.574, R^2^ = 0.329, Adjusted R^2^ = 0.181; F = 2.220, *p* = 0.003; Predictors 3rd month: R = 0.617, R^2^ = 0.381, Adjusted R^2^ = 0.244; F = 2.780, *p* < 0.001.

### Ethical approval

This study was approved by the Regional and Institutional Research Ethics Committee of the Clinical Centre of the University of Pécs (ID: 7839-PTE 2019, 07/06/2019). The study was performed in accordance with relevant guidelines and regulations and the Declaration of Helsinki. Written informed consent was obtained to participate in the study.

## Results

In 2019, a total of 439 hip replacement surgeries were performed in the public and 373 surgeries in the private healthcare sector, from which 212 patients were assessed during our study. Among the 212 patients, 128 people (60.38%) were included in our research, 60 people from the public healthcare, 68 people from private healthcare. We evaluated 26.11% of the total number of patients operated on in that year, and 15.76% were included in the study (13.67% in public hospital, 18.23% in private hospital). The main reason for dropout was the incomplete completion of the questionnaires and the failure to return the follow-up questionnaires.

### Sociodemographic data

Table 1 summarizes the sociodemographic data of the participants. The age distribution shows that the highest proportion of patients aged 65 years and over were present in both hospitals, but a significantly higher proportion of this age group was found in the public hospital. Body mass index (BMI) was categorised according to the World Health Organisation (WHO) recommendations: < 18.5 as underweight, 18.5–24.9 as normal, 25–29.9 as overweight, and > 30 as obesity. The BMI analysis showed that the highest proportion of patients were overweight and obese. There was no difference between public and private hospitals in this aspect. From patients of public hospital, it is significantly higher for those from the village (public: 33.33%, private: 17.65%, p = 0.041), with primary education (public: 21.67%, private: 2.94%, p = 0.001), the proportion of retired patients (public: 75.0%; private: 47.06%; p = 0.001), while in the private health sector it is higher for those living in the county seat, in the capital city, with higher education (public: 21.67%, private: 55.88%, p < 0.001), doing intellectual work (public: 8.33%; private: 39.71%; p < 0.001) and are private entrepreneur (public: 0%; private: 33.82%; p < 0.001).

### Surgical data

Examining the reason for the surgery, we can see that the respondents in both groups underwent surgery mainly due to the diagnosis of hip osteoarthritis (coxarthrosis) (public: 81.67%; private: 85.29%). The average waiting time in public healthcare was 5–6 months, while in private healthcare it was 3 weeks. The type of surgical procedure and type of explorations in the investigated groups differs significantly from one another because in public healthcare, the traditional procedure (muscle transection) with anterolateral exploration is preferred, while in the case of the private system, patients are only operated with a minimally invasive (muscle displacement), anterior exploration surgical technique (p < 0.001). In public healthcare, cemented prostheses are significantly higher, while in private healthcare, the proportion of non-cemented prostheses is significantly higher (p < 0.001). Table 2 summarizes the surgical data.

### Quality of life

In Table 3, we can see the changes in the results of the Oxford Hip Score and SF-36 questionnaires depending on the time since the surgery, broken down by care areas.

**Figure 1:**
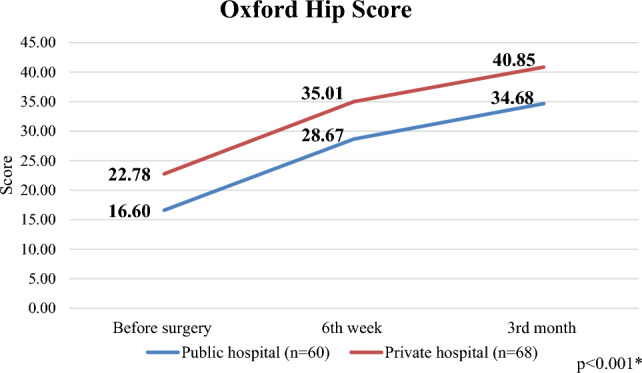
Changes in the Oxford Hip Score (OHS) in public and private hospitals.

The Oxford Hip Score showed significant improvement in both public and private healthcare, comparing preoperative, 6-week and 3-month postoperative results. In the case of public healthcare patients, the values increased from 16.60 points to 28.67 points after 6 weeks, and then to 34.68 points in the 3rd month after surgery (*p* < 0.001). The patients of private healthcare had 22.78 points before surgery, which increased to 35.01 points by the 6th week and 40.85 points by the 3rd month (*p* < 0.001) (Fig. 1). When comparing the results of the two groups, a significant difference can be seen between the preoperative, 6-week and 3-month results (*p* < 0.001). However, comparing the degree of improvement in the quality of life during the 3-month period, according to the OHS questionnaire, there is no difference between the two healthcare sectors (*p* = 0.985).

**Figure 2:**
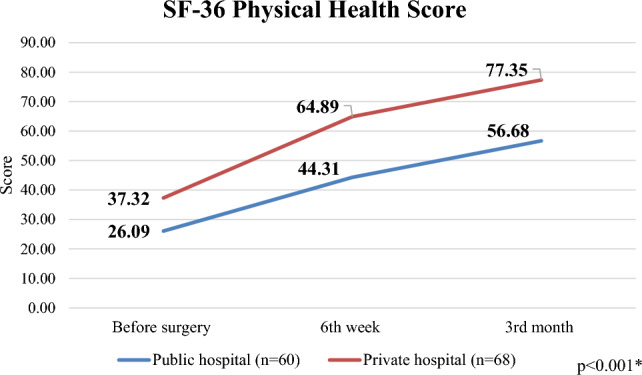
Changes in the SF-36 Physical Health Score in public and private hospitals.

According to the SF-36 questionnaire, the *physical health* score of public healthcare patients increased from 26.09 points preoperatively to 44.31 points after 6 weeks, and then to 56.68 points by the 3rd month (*p* < 0.001), while the score of private healthcare patients increased from 37.32 points to 64.89 points (6th week) and to 77.35 points (3rd month) (*p* < 0.001) (Fig. 2). A significant difference can be seen between the preoperative, 6-week and 3-month follow-up results of the two care areas (*p* < 0.001). Regarding the total improvement measured during the survey period, a significant difference can be seen between the groups, on the basis of which the patients of the public hospital achieved a greater improvement (*p* = 0.027).

In the *mental health* score, only the patients of the private health sector showed a significant improvement, as their result increased from 68.63 points preoperatively to 81.09 points by the 6th week and then to 86.18 points by the 3rd month (*p* < 0.001). On the other hand, the preoperative score of 66.85 points for the patients of the public hospital increased to only 67.25 (6th week) and then 73.86 points (3rd month) (*p* = 0.075). Comparing the results of the two groups there is no significant difference between them in the preoperative values, but for the 6th week (*p* < 0.001) and the 3rd month (*p* < 0.001) a significant difference between the public and private healthcare is shown. In the improvement of the mental quality of life during the entire survey period, we observed a significant difference when comparing the results of public and private healthcare, but unlike physical health, here the patients of private healthcare achieved a greater degree of improvement (*p* = 0.015).

### Subgroup analysis: sociodemographic characteristics and quality of life

In the second part of our research, we performed a subgroup analysis examining how the quality of life regarding public and private healthcare patients according to the Oxford Hip Score questionnaire changed depending on sociodemographic characteristics for the 3^rd^ postoperative month (Table 4).

When broken down by sex, a significant difference can be seen in the preoperative values of men and women, which balances out by the 3rd month. The change between the results before surgery and the follow-up is significant in all cases (*p* < 0.001). In terms of improvement within the group, only private healthcare showed a significant result (*p* = 0.001). When comparing age groups and quality of life scores, no significant differences were found either within or between groups, but there was a significant improvement in baseline quality of life for all age groups by the third month. In the case of BMI, all baseline quality of life scores also improved significantly. There was also a significant difference in the extent of improvement in quality of life between patients with normal BMI in public and private hospitals. For private patients, a significant difference was found in the 3-month quality of life outcomes of patients with different BMI categories, as well as in the extent of improvement in quality of life for private patients. We did not find any difference between the quality of life regarding different age groups, people living in different residences, and individuals living in different marital statuses before the operation and in the 3rd month after the operation, however, during the follow-up, the quality of life regarding all groups showed a significant improvement.

The quality of life before and after surgery was the same among those with various educational qualifications, however, the quality of life of the patients in public healthcare showed a significant difference by the 3rd month according to education (*p* = 0.039). The improvement was significant in all cases by the third month. While there was no significant difference in the degree of improvement in the case of public hospital (*p* = 0.231), there was a significant difference in private hospital (*p* = 0.021). Overall, we could see a significant difference between the improvement in the quality of life considering patients with different educational qualifications in public and private healthcare (*p* = 0.010).

The results of the pre- and postoperative surveys did not differ according to occupation, however, after the follow-up period, light physical work and retired patients of the public hospital, as well as intellectual, light physical work and retired patients of the private hospital, achieved a significant improvement. In the case of those performing light physical work, we can see a significant difference in the degree of improvement between public and private healthcare patients (*p* = 0.026). The improvement in the quality of life regarding patients in the public sector differed depending on the occupations (*p* = 0.049), but this cannot be said for private patients (*p* = 0.804). The quality of life considering those employed in various employment relationships—with the exception of public hospital government employee patients—showed a significant improvement in the 3rd postoperative month.

### Surgical characteristics and quality of life

As part of the second half of our research, we examined the quality-of-life scores of the Oxford Hip Score questionnaire as a function of the surgical characteristics, both in public and private healthcare (Table 5).

In the case of most surgical indications, the quality-of-life scores showed a significant increase in the postoperative 3rd month. In the case of public healthcare, preoperative results differed depending on the indication (*p* = 0.001), and the total improvement within the group also differed between them (*p* = 0.024).

Due to the fundamental difference in the practical preference of the surgical procedure and surgical exploration, we were mainly able to determine the improvement in quality of life over time, which was significant for all types by the third month after surgery (*p* < 0.001).

Depending on the type of fixation, the patients of the public hospital had different preoperative quality-of-life scores (*p* = 0.015), which levelled off by the 3rd month (*p* = 0.121). The quality of life measured during the follow-up showed a significant improvement in both healthcare sectors (*p* < 0.001).

According to the type of anaesthesia, we cannot see any difference in the quality-of-life scores measured in each survey period, however, by the 3rd month, we saw a significant improvement for all types. Regardless of the use of the drain and blood transfusion, all patients achieved a significant improvement by the third month after the operation (*p* < 0.001).

### Association between quality-of-life score*s,* socioeconomic data and type of hospital

The association between quality-of-life outcomes (OHS), socioeconomic data and type of hospital was analysed using multivariate linear regression analysis (Table 6). The subcategories were compared for each variable and the reference value was indicated. Comparing the Oxford Hip Score results at postoperative 6th week with the provided variables (sex, age group, BMI, residence, education, marital status, occupation, employee status, type of hospital), we found that marital status and type of hospital significantly influenced the quality-of-life score. Patients in a single marital status had higher quality of life scores than married (*β* = − 7.443, *p* = 0.034), in a relationship (*β* = − 8.695, *p* = 0.046), divorced (*β* = − 10.934, *p* = 0.006) and widows (*β* = − 10.539, *p* = 0.016). Furthermore, patients in the private hospital also had a higher quality of life score than patients in the public hospital (*β* = 3.968, *p* = 0.036). Comparing the Oxford Hip Score at postoperative 3rd month, we found that marital status, occupation and type of hospital were significantly associated with quality of life. Patients in single marital status had significantly higher quality of life scores than divorced (*β* = − 10.305, *p* = 0.006) and widowed patients (*β* = − 10.039, *p* = 0.014). Compared to light physical workers, the quality-of-life scores of unemployed and disabled people belonging to “Other” category were significantly lower (β = − 12.817, *p* = 0.035). Private patients also had higher quality-of-life scores at the 3rd month than patients at the public hospital (*β* = 5.007, *p* = 0.005).

## Discussion

We examined the impact of hip replacement surgery on quality of life, as well as the evolution of quality of life according to sociodemographic and surgical data in the public and private hospitals in Hungary. We first examined the patients’ sociodemographic and surgery-related data, where we found significant differences between the two groups. From patients of public hospital, it is significantly higher for those aged > 65, from the village, with primary education, proportion of retired patients, while in private hospital it is higher for those living in the county seat, in the capital city, with higher education, doing intellectual work and are private entrepreneur. Rana et al*.*^27^ obtained similar results to our study in their analysis of healthcare utilisation behaviour of patients with private health insurance in Australia in 2020. Similar to the sociodemographic characteristics, the surgical data also showed significant differences between patients in the two hospitals. While the public hospital operated with a conventional procedure, anterolateral exploration and 60% cemented prosthesis, the private hospital operated only with minimally invasive procedure, anterior exploration and 92.65% non-cemented fixation. The difference between the surgical procedures used can be explained in several ways (e.g. surgeon/ hospital preference, surgeon experience, patient factors)^28^. In private hospital, the role of marketing is also significant, as minimally invasive technique may achieve early mobilisation, shorter length of stay and better short-term functional outcomes. However, in the investigated public hospital, a conventional procedure is used, as current meta-analyses of randomized controlled trials confirm that there is no clear clinical benefit of minimally invasive procedure^29,30^.

The main aim of the study was to compare the quality of life between the patients of the two healthcare sectors. Patients in both groups achieved significant improvement in the OHS questionnaire and SF-36 physical health scores 6 weeks and 3 months after surgery. Only private patients achieved a significant increase in the SF-36 mental health score. In the case of all quality-of-life indicators, the patients of the public hospital had significantly lower preoperative scores, however, considering the entire study period, according to the OHS questionnaire, there was no difference in the improvement of the quality of life between the two hospitals. In addition to socio-economic factors, as many studies have shown, the longer waiting time may also have contributed to the worse preoperative quality of life of patients provided with public healthcare^31,32^. Similar to Fitzpatrick et al*.*^33^ research, we also experienced longer waiting times in the case of public healthcare.

In the second half of our research, we performed a comparison of the sociodemographic and surgical data, as well as the Oxford Hip Score preoperative and postoperative 3-month quality of life scores. Despite the different sociodemographic and surgical characteristics, all patients achieved a significant improvement based on quality-of-life scores by the end of the follow-up period. In the case of private patients, we observed a difference in the improvement of the quality of life between sexes and according to educational level. Between the two hospitals, the change in the quality of life of those with different educational qualifications was not the same either. From the side of the public hospital, we found that patients with different occupations and operated with different surgical indications did not achieve the same level of improvement in quality-of-life results. We should consider that the general health status of the Hungarian population is worse compared to Western-European or North-American countries^34–36^. Our multivariate linear regression analysis found that quality-of-life scores were associated with marital status, occupation, and type of hospital where surgery was performed.

In the course of our previous pilot research, a short-term (6-week) quality of life examination of public and private healthcare patients was conducted, based on which it was already shown that there is a significant difference between the sociodemographic and surgical characteristics of hip replacement patients. Despite all these differences, already 6 weeks after the operation, we experienced a significant improvement in the postoperative quality of life (with the help of the SF-36 and OHS questionnaire), both in the case of public and private healthcare. We found a significant difference in the utilisation of rehabilitation between hospitals even at week 6. While 90.67% of patients in the public hospital received rehabilitation, only 59.55% of private patients attended rehabilitation (*p* < 0.001)^22,37,38^. In private hospitals, patients can leave the hospital quickly and recover their mobility quickly. Taking into account personal health and safety, it would also be important for patients undergoing private surgery to be properly rehabilitated and followed up more effectively. The present study also showed that the rehabilitation utilisation rate differs significantly between hip replacement patients operated in public and private hospitals. While 93.33% of public patients received rehabilitation after surgery, only 60.29% of private patients used it (*p* < 0.001). While 88.33% of public patients received regular care and half of the patients started rehabilitation immediately after surgery, only 51.47% of private patients received regular care and only 17.65% started treatment immediately (*p* < 0.001). Public patients mainly visit public rehabilitation institutions or use public home care, private patients mainly choose public and private home care (*p* < 0.001).

In a recent study, Moarrefzadeh et al*.*^39^ investigated the postoperative quality of life in an elderly Iranian population. Using the SF-36 questionnaire there was a significant improvement in the scores both 6 months and 12 months after the surgery, where the physical health scores in the first period and the mental health scores in the second period also showed a significant increase. In contrast, Balik et al*.*^40^ observed a significant improvement in SF-36 physical health scores and stagnation in mental health scores at 6 and 12 weeks of follow-up. During our study, a significant improvement in physical health could be demonstrated during the 3-month period, however, the mental health scores did not change after hip replacement surgery of patients in public hospital. Our suggestion to the public hospitals would be that in addition to improving physical health, it is also necessary to focus on improving mental health, which has resulted in poorer outcomes in their case.

The change in the quality of life according to the healthcare sector was also investigated by Heath et al*.*^41^. According to their research conducted in Australian hospitals, there was a significant relationship between the type of care system and the OHS score at a 6-month follow-up. The preoperative OHS score—similarly to our own research—was significantly higher in patients from private healthcare. In their 2019 research, Naylor et al.^42^ published preoperative quality of life scores similar to our study based on the OHS questionnaire. According to their results, public healthcare patients scored an average of 17 points and private patients scored 23.6 points (*p* < 0.001), while in our own research, public patients scored 16.6 points and private patients scored 22.8 points (*p* < 0.001). Adie et al*.* used the OHS and SF-36 questionnaires to assess the quality of life of hip replacement patients before surgery and 6 and 12 months after surgery^43^. According to their results, patients from the public and private care sectors both achieved a significant improvement in postoperative quality of life, and contrary to our results, there was no difference between them in the degree of improvement in quality of life. Private patients had higher preoperative SF-36 mental health and OHS scores and equal preoperative SF-36 physical health scores. In our research, the SF-36 physical health and OHS scores showed higher values in private healthcare patients, and the mental health score was almost the same. Similar to our own results, in all cases the patients of private hospital had a better quality of life, however, the scores we received were higher in most cases. Agerholm et al*.* compared Swedish integrated care and standard care in terms of patient-reported outcomes (EQ VAS score, EQ-5D-3L, hip pain VAS score). Their results showed that 1 year after total hip replacement, the health status of patients in integrated care did not differ significantly from patients in standard care^44^.

Mannion et al*.* compared the OHS quality of life scores of men and women at one-year follow-up. Similar to our results, women experienced a lower preoperative quality of life, which equalized between the two sexes during the follow-up^45^. In an age-based comparison, Umehara et al*.*^46^ showed a greater improvement in the case of younger patients using the SF-36 questionnaire, however, the results we obtained could not confirm this based on the OHS questionnaire, as there was no significant difference in the degree of improvement in the quality of life of patients of different ages. According to Peters et al*.*^47^, young female patients with a high BMI achieve a greater improvement in postoperative quality of life results, in our case this was only confirmed from the side of private female patients. The association of quality of life with sociodemographic data was also investigated by Szende et al*.*^48^ using the EQ-5D-3L questionnaire. Their results showed that age, education level and income have a significant impact on self-reported health.

Vučković et al*.*^49^ used the SF-36 questionnaire to examine the quality of life of patients operated on with a traditional and minimally invasive procedure, according to which the intervention performed with a minimally invasive procedure achieved significantly better results in 5 out of 8 quality of life dimensions. In our own study, the minimally invasive procedure was only more beneficial in the case of 2 dimensions. Galmiche et al.^14^ found no significant difference in quality-of-life scores in the case of anterior, posterior and lateral exploration. Castioni et al.^50^ compared lateral and posterior exploration in terms of quality of life, and the scales used showed better results for posterior exploration. In our case, the differences between anterior and anterolateral exploration were examined, in which higher scores were shown in the case of anterior exploration, similar to the results published by Amlie et al. in 2014^51^. Jin et al. in a recent study in 2023 compared direct anterior approach with posterolateral approach in simultaneous bilateral total hip arthroplasty, where similarly as in our study, patients operated on with direct anterior approach had lower transfusion rates (18% vs. 36%; in our study: 0% vs. 31.03%), shorter length of stay (5.12 vs 6.4 days; in our study: 2.03 vs. 6.07 days) and smaller limb length differences (2.1 vs. 3.8 mm; in our study: 1.3 vs. 3.4 mm) than patients operated on with posterolateral approach. They also had a shorter incision length and reported less pain and better functional outcomes postoperatively^52^. In the study of quality of life and fixation of the prosthesis, Bagarić et al*.* recorded better indicators of non-cemented prosthesis. In our case, although the non-cemented prosthesis had higher scores, there was no difference in the degree of improvement compared to the cemented fixation^53^.

The main limitation of our research is that it covered a short period of 3 months. For the sake of long-term results, it is definitely advisable to extend the follow-up period. Our results were compiled from subjective data reported by patients, the quality-of-life results were not compared with objective imaging diagnostics and preoperative joint indicators. An additional limitation of our research is that only two clinics in Pécs participated in the study, the results cannot be generalized to the whole of Hungary, our results can only be inferred. Limitations may include a low number of patients. 15.76% of all patients operated completed the questionnaires, which may lead to bias. Furthermore, we would like to emphasize that the study analysed two different populations, as it compared patients from public and private hospitals, which also means that two different surgical procedures (minimally invasive versus conventional) and exploration (anterior versus anterolateral exploration) were considered. We intend to continue our research in the future, where, taking into account the mentioned limitations, we would include additional test variables, as well as expand the number of patients, the test period, and study locations with more clinics in Hungary.

In summary, it can be said that despite the different socio-demographic characteristics, the different surgical procedure, exploration and fixation, in the case of patients who underwent hip replacement surgery, a significant improvement in the postoperative quality of life results was shown in terms of both healthcare sectors, comparing the pre-operative scores 6 weeks and 3 months after the surgery. The change in the Oxford Hip Score did not show a significant difference between the two hospitals, the scores increased in the same way in the postoperative period, however, based on the SF-36 questionnaire, we could see a significant difference in the postoperative improvement of the quality of life. While the SF-36 physical health score showed a higher improvement for public patients (*p* = 0.027), the mental health score showed a higher improvement for private patients (*p* = 0.015). We finally found that postoperative quality-of-life scores were associated with marital status, occupation, and type of hospital where surgery was performed. Although the surgical procedures and exploration used in public and private hospitals are different, both can significantly improve the quality of life. The appropriate decision on the type of surgery is mostly based on the institution’s or surgeon’s decision, experience and facilities, but it should always be adapted to the patient’s health, aims and possibilities. In order to make the most appropriate clinical decisions, to investigate the effectiveness of different surgical procedures, to prove the clinical relevance of the results and to achieve them, long-term, multicentre studies and meta-analyses are required.

## Data Availability

The datasets generated during and/or analysed during the current study are available from the corresponding author on reasonable request.
